# (6*RS*,9*SR*)-6,7-Dibromo-1,2,3,4-tetra­hydro-1,4-methano­anthracene

**DOI:** 10.1107/S1600536811013572

**Published:** 2011-04-16

**Authors:** Kew-Yu Chen, Ming-Jen Chang, Tzu-Chien Fang

**Affiliations:** a Department of Chemical Engineering, Feng Chia University, 40724 Taichung, Taiwan

## Abstract

The title compound, C_15_H_12_Br_2_, comprises a norbornane unit having a dibromo­naphthalene ring fused on one side. Both Br atoms are twisted slightly out of the plane of the naphthalene ring system with a Br—C—C—Br torsion angle of 5.3 (5)°. In the crystal, mol­ecules are linked by weak inter­molecular C—H⋯Br hydrogen bonds, forming an infinite *C*(9) chain along [110].

## Related literature

For the spectroscopy of the title compound and its preparation, see: Chen *et al.* (2006 ▶). For the spectroscopy and electronic device applications of rigid oligo-norbornyl compounds, see: Chen *et al.* (2002 ▶); Chow *et al.* (2005 ▶); Lewis *et al.* (1997 ▶); Roest *et al.* (1996 ▶). For related structures, see: Çelik *et al.* (2006 ▶); Chiou *et al.* (2001 ▶); Chow *et al.* (1999 ▶); Lough *et al.* (2006 ▶). For the C—H⋯Br hydrogen bond, see: Desiraju & Steiner (2001 ▶); Farrugia *et al.* (2007 ▶); Kuś & Jones (2003 ▶); Yang *et al.* (2007 ▶). For puckering parameters, see: Cremer & Pople (1975 ▶). For graph-set theory, see: Bernstein *et al.* (1995 ▶).
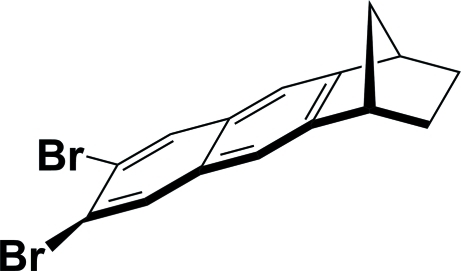


## Experimental

### 

#### Crystal data

C_15_H_12_Br_2_
*M* *_r_* = 352.07
Monoclinic, 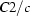

*a* = 23.437 (3) Å
*b* = 6.3565 (8) Å
*c* = 18.416 (2) Å
β = 111.781 (2)°
*V* = 2547.6 (6) Å^3^
*Z* = 8
Mo *K*α radiation
μ = 6.34 mm^−1^
*T* = 297 K
0.56 × 0.48 × 0.20 mm

#### Data collection

Bruker SMART CCD area-detector diffractometer
Absorption correction: multi-scan (*SADABS*; Bruker, 2001 ▶) *T*_min_ = 0.399, *T*_max_ = 1.000
6895 measured reflections
2501 independent reflections
1817 reflections with *I* > 2σ(*I*)
*R*_int_ = 0.058

#### Refinement

*R*[*F* ^2^ > 2σ(*F* ^2^)] = 0.055
*wR*(*F* ^2^) = 0.144
*S* = 0.96
2501 reflections
154 parameters
H-atom parameters constrained
Δρ_max_ = 1.12 e Å^−3^
Δρ_min_ = −1.09 e Å^−3^

### 

Data collection: *SMART* (Bruker, 2001 ▶); cell refinement: *SAINT* (Bruker, 2001 ▶); data reduction: *SAINT*; program(s) used to solve structure: *SHELXS97* (Sheldrick, 2008 ▶); program(s) used to refine structure: *SHELXL97* (Sheldrick, 2008 ▶); molecular graphics: *ORTEP-3 for Windows* (Farrugia, 1997 ▶); software used to prepare material for publication: *WinGX* (Farrugia, 1999 ▶).

## Supplementary Material

Crystal structure: contains datablocks I, global. DOI: 10.1107/S1600536811013572/nr2004sup1.cif

Structure factors: contains datablocks I. DOI: 10.1107/S1600536811013572/nr2004Isup2.hkl

Additional supplementary materials:  crystallographic information; 3D view; checkCIF report

## Figures and Tables

**Table 1:** Hydrogen-bond geometry (Å, °)

*D*—H⋯*A*	*D*—H	H⋯*A*	*D*⋯*A*	*D*—H⋯*A*
C8—H8*A*⋯Br2^i^	0.97	3.00 (1)	3.843 (16)	146 (1)

Symmetry code: (i) 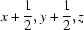.

**Fig. 1.**
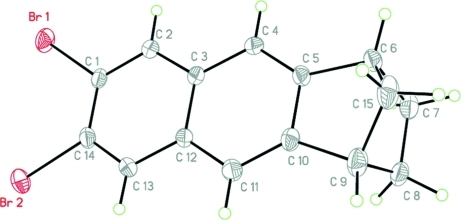
The molecular structure of the title compound, showing 50% probability displacement ellipsoids.

**Fig. 2.**
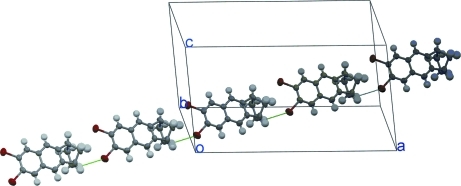
A section of the crystal packing of the title compound, viewed along the b axis.

## supplementary crystallographic information

### Comment

Electron donor (D)–acceptor (A) chromophores linked by rigid, covalent spacers (S), forming D–S–A dyads, have attracted considerable attention due to their potential applications in the design of molecular devices (Lewis *et al.*, 1997; Roest *et al.*, 1996). Numerous types of spacers have been reported (Chiou *et al.*, 2001; Chow *et al.*, 1999). However, rigid linear rod-shaped structures are not commonly seen. The highly symmetrical structures reduce the complexity due to the constraint of geometrical and conformational variations. The rates of photoinduced electron transfer reactions across linearly fused oligo-norbornyl spacer groups have been estimated (Chen *et al.*, 2002; Chow *et al.*, 2005). The ET rates were found to correlate well with both D–A distance and solvent polarities. Atoms C6 and C9 of the title compound are chiral centers, but their relative configurations are opposite (6*R*,9S or 6S,9*R*). The racemate was prepared as a model compound for investigations of the intramolecular electron transfer reactions (Chen *et al.*, 2006).

The *ORTEP* diagram of the title compound is shown in Figure 1. The puckering parameters (Cremer & Pople, 1975) of the five-membered rings *A* (C5/C6/C15/C9/C10) and *B* (C6–C9/C15) are *Q*_2_ = 0.560 (6)Å and φ_2_ = 71.0 (5)°, and *Q*_2_ = 0.602 (6)Å and φ_2_ = 144.7 (6)°, respectively. These results are slightly different from those of previous studies on other norbornane derivatives (Çelik, *et al.*, 2006; Lough, *et al.*, 2006). In addition, the naphthalene ring is essentially planar with a maximum deviation of 0.052 (2)Å for atom C5. Whereas both bromine atoms are slightly twisted out of the plane of the naphthalene ring (5.3 (5)° of Br1—C1—C14—Br2, Table 1). In the crystal structure (Figure 2), the molecules are linked by weak intermolecular C—H···Br (2.998 (2)Å of C8—H8A···Br2 distance and 146 (1)° of C8—H8A—Br2, Table 2) hydrogen bonds (Desiraju *et al.*, 2001; Farrugia *et al.*, 2007; Kuś *et al.*, 2003; Yang *et al.*, 2007) to form an infinite two-dimensional chain, generating a C(9) motif (Bernstein *et al.*, 1995).

### Experimental

A mixture of α,α,α',α'-4,5- hexabromo-*o*-xylene (4.3 mmol), norbornene (4.3 mmol), sodium iodide (30 mmol), and dry DMF (50 ml) was stirred at 65 *^o^*C for 24 h. The reaction mixture was poured into cold water (350 ml) containing sodium bisfulfite (5.0 g). The yellow precipitate was purified by chromatography (silica gel column, hexane:ethyl acetate = 6:1) and finally by recrystallization. Colorless needle-shaped crystals suitable for the crystallographic studies reported here were isolated over a period of five weeks by slow evaporation from a chloroform solution.

### Refinement

The C bound H atoms positioned geometrically (C—H = 0.93–0.98 Å) and allowed to ride on their parent atoms, with *U*_iso_(H) = 1.2*U*_eq_(C)].

### Crystal data

**Table:**

C_15_H_12_Br_2_	*F*(000) = 1376
*M**_r_* = 352.07	*D*_x_ = 1.836 Mg m^−^^3^
Monoclinic, *C*2/*c*	Mo *K*α radiation, λ = 0.71073 Å
Hall symbol: -C 2yc	Cell parameters from 2464 reflections
*a* = 23.437 (3) Å	θ = 3.3–25.5°
*b* = 6.3565 (8) Å	µ = 6.34 mm^−^^1^
*c* = 18.416 (2) Å	*T* = 297 K
β = 111.781 (2)°	Parallelepiped, colorless
*V* = 2547.6 (6) Å^3^	0.56 × 0.48 × 0.20 mm
*Z* = 8	

### Data collection

**Table:**

Bruker SMART CCD area-detector diffractometer	2501 independent reflections
Radiation source: fine-focus sealed tube	1817 reflections with *I* > 2σ(*I*)
graphite	*R*_int_ = 0.058
φ and ω scans	θ_max_ = 26.1°, θ_min_ = 1.9°
Absorption correction: multi-scan (*SADABS*; Bruker, 2001)	*h* = −19→28
*T*_min_ = 0.399, *T*_max_ = 1.000	*k* = −7→7
6895 measured reflections	*l* = −22→21

### Refinement

**Table:**

Refinement on *F*^2^	Primary atom site location: structure-invariant direct methods
Least-squares matrix: full	Secondary atom site location: difference Fourier map
*R*[*F*^2^ > 2σ(*F*^2^)] = 0.055	Hydrogen site location: inferred from neighbouring sites
*wR*(*F*^2^) = 0.144	H-atom parameters constrained
*S* = 0.96	*w* = 1/[σ^2^(*F*_o_^2^) + (0.095*P*)^2^] where *P* = (*F*_o_^2^ + 2*F*_c_^2^)/3
2501 reflections	(Δ/σ)_max_ < 0.001
154 parameters	Δρ_max_ = 1.12 e Å^−^^3^
0 restraints	Δρ_min_ = −1.09 e Å^−^^3^

### Special details

**Table:**

Geometry. All e.s.d.'s (except the e.s.d. in the dihedral angle between two l.s. planes) are estimated using the full covariance matrix. The cell e.s.d.'s are taken into account individually in the estimation of e.s.d.'s in distances, angles and torsion angles; correlations between e.s.d.'s in cell parameters are only used when they are defined by crystal symmetry. An approximate (isotropic) treatment of cell e.s.d.'s is used for estimating e.s.d.'s involving l.s. planes.
Refinement. Refinement of *F*^2^ against ALL reflections. The weighted *R*-factor *wR* and goodness of fit *S* are based on *F*^2^, conventional *R*-factors *R* are based on *F*, with *F* set to zero for negative *F*^2^. The threshold expression of *F*^2^ > σ(*F*^2^) is used only for calculating *R*-factors(gt) *etc*. and is not relevant to the choice of reflections for refinement. *R*-factors based on *F*^2^ are statistically about twice as large as those based on *F*, and *R*- factors based on ALL data will be even larger.

### Fractional atomic coordinates and isotropic or equivalent isotropic displacement parameters (Å^2^)

**Table:**

	*x*	*y*	*z*	*U*_iso_*/*U*_eq_	
Br1	0.02569 (2)	0.67170 (9)	0.16329 (3)	0.0637 (2)	
Br2	0.02816 (3)	0.21416 (10)	0.07270 (4)	0.0725 (3)	
C1	0.0959 (2)	0.5881 (7)	0.1431 (2)	0.0420 (10)	
C2	0.1462 (2)	0.7148 (7)	0.1649 (2)	0.0451 (10)	
H2A	0.1447	0.8436	0.1881	0.054*	
C3	0.2004 (2)	0.6583 (7)	0.1536 (2)	0.0406 (10)	
C4	0.2517 (2)	0.7938 (7)	0.1720 (2)	0.0451 (11)	
H4A	0.2510	0.9253	0.1938	0.054*	
C5	0.3020 (2)	0.7309 (7)	0.1576 (2)	0.0439 (10)	
C6	0.3608 (2)	0.8382 (8)	0.1631 (3)	0.0545 (13)	
H6A	0.3726	0.9605	0.1979	0.065*	
C7	0.3561 (2)	0.8766 (9)	0.0789 (3)	0.0583 (13)	
H7A	0.3178	0.9466	0.0488	0.070*	
H7B	0.3900	0.9621	0.0779	0.070*	
C8	0.3584 (2)	0.6567 (8)	0.0465 (3)	0.0595 (14)	
H8A	0.3932	0.6426	0.0302	0.071*	
H8B	0.3209	0.6252	0.0025	0.071*	
C9	0.3656 (2)	0.5124 (9)	0.1171 (3)	0.0612 (13)	
H9A	0.3814	0.3705	0.1151	0.073*	
C10	0.3047 (2)	0.5253 (7)	0.1275 (3)	0.0454 (10)	
C11	0.2564 (2)	0.3915 (8)	0.1093 (3)	0.0513 (11)	
H11A	0.2586	0.2587	0.0893	0.062*	
C12	0.2025 (2)	0.4553 (6)	0.1210 (2)	0.0387 (9)	
C13	0.1496 (2)	0.3277 (7)	0.0989 (3)	0.0479 (11)	
H13A	0.1504	0.1961	0.0772	0.057*	
C14	0.0971 (2)	0.3921 (7)	0.1085 (2)	0.0454 (10)	
C15	0.4058 (2)	0.6541 (10)	0.1856 (3)	0.0692 (16)	
H15A	0.4115	0.5958	0.2365	0.083*	
H15B	0.4452	0.6885	0.1826	0.083*	

### Atomic displacement parameters (Å^2^)

**Table:**

	*U*^11^	*U*^22^	*U*^33^	*U*^12^	*U*^13^	*U*^23^
Br1	0.0470 (3)	0.0745 (4)	0.0825 (4)	−0.0007 (3)	0.0389 (3)	0.0001 (3)
Br2	0.0554 (4)	0.0786 (4)	0.0901 (5)	−0.0332 (3)	0.0345 (3)	−0.0187 (3)
C1	0.038 (2)	0.052 (3)	0.041 (2)	−0.005 (2)	0.021 (2)	0.0023 (18)
C2	0.046 (3)	0.048 (3)	0.048 (3)	−0.007 (2)	0.025 (2)	−0.0068 (19)
C3	0.043 (2)	0.045 (2)	0.038 (2)	−0.006 (2)	0.020 (2)	−0.0012 (18)
C4	0.046 (3)	0.054 (3)	0.038 (2)	−0.009 (2)	0.019 (2)	−0.0092 (18)
C5	0.037 (2)	0.056 (3)	0.038 (2)	−0.013 (2)	0.013 (2)	−0.0047 (19)
C6	0.044 (3)	0.072 (3)	0.049 (3)	−0.023 (2)	0.018 (2)	−0.011 (2)
C7	0.049 (3)	0.072 (3)	0.058 (3)	−0.009 (3)	0.024 (2)	0.008 (2)
C8	0.041 (3)	0.087 (4)	0.059 (3)	−0.011 (3)	0.028 (2)	−0.011 (3)
C9	0.041 (3)	0.065 (3)	0.084 (4)	0.004 (3)	0.031 (3)	0.007 (3)
C10	0.034 (2)	0.056 (3)	0.048 (2)	−0.001 (2)	0.017 (2)	0.003 (2)
C11	0.050 (3)	0.042 (2)	0.068 (3)	−0.001 (2)	0.030 (2)	−0.003 (2)
C12	0.039 (2)	0.041 (2)	0.039 (2)	−0.0049 (19)	0.0171 (19)	−0.0002 (17)
C13	0.052 (3)	0.040 (2)	0.059 (3)	−0.011 (2)	0.029 (2)	−0.0076 (19)
C14	0.041 (2)	0.052 (3)	0.044 (2)	−0.013 (2)	0.017 (2)	0.0022 (19)
C15	0.036 (3)	0.107 (5)	0.061 (3)	−0.003 (3)	0.013 (2)	0.019 (3)

### Geometric parameters (Å, °)

**Table:**

Br1—C1	1.892 (4)	C7—H7A	0.9700
Br2—C14	1.881 (4)	C7—H7B	0.9700
C1—C2	1.359 (6)	C8—C9	1.548 (7)
C1—C14	1.405 (6)	C8—H8A	0.9700
C2—C3	1.407 (6)	C8—H8B	0.9700
C2—H2A	0.9300	C9—C10	1.512 (6)
C3—C12	1.432 (6)	C9—C15	1.552 (8)
C3—C4	1.415 (6)	C9—H9A	0.9800
C4—C5	1.363 (6)	C10—C11	1.355 (7)
C4—H4A	0.9300	C11—C12	1.417 (6)
C5—C10	1.430 (7)	C11—H11A	0.9300
C5—C6	1.506 (6)	C12—C13	1.409 (6)
C6—C15	1.526 (8)	C13—C14	1.368 (7)
C6—C7	1.533 (6)	C13—H13A	0.9300
C6—H6A	0.9800	C15—H15A	0.9700
C7—C8	1.529 (7)	C15—H15B	0.9700
			
C2—C1—C14	119.8 (4)	C7—C8—H8B	111.2
C2—C1—Br1	119.8 (3)	C9—C8—H8B	111.2
C14—C1—Br1	120.3 (3)	H8A—C8—H8B	109.1
C1—C2—C3	122.4 (4)	C10—C9—C8	105.1 (4)
C1—C2—H2A	118.8	C10—C9—C15	100.5 (4)
C3—C2—H2A	118.8	C8—C9—C15	100.6 (4)
C12—C3—C4	119.3 (4)	C10—C9—H9A	116.1
C12—C3—C2	117.8 (4)	C8—C9—H9A	116.1
C4—C3—C2	122.9 (4)	C15—C9—H9A	116.1
C5—C4—C3	119.7 (4)	C11—C10—C5	121.1 (4)
C5—C4—H4A	120.1	C11—C10—C9	132.6 (5)
C3—C4—H4A	120.1	C5—C10—C9	106.1 (4)
C4—C5—C10	120.6 (4)	C10—C11—C12	119.5 (4)
C4—C5—C6	133.7 (4)	C10—C11—H11A	120.2
C10—C5—C6	105.7 (4)	C12—C11—H11A	120.2
C5—C6—C15	101.2 (4)	C11—C12—C3	119.6 (4)
C5—C6—C7	106.4 (4)	C11—C12—C13	122.0 (4)
C15—C6—C7	100.4 (4)	C3—C12—C13	118.4 (4)
C5—C6—H6A	115.6	C14—C13—C12	121.8 (4)
C15—C6—H6A	115.6	C14—C13—H13A	119.1
C7—C6—H6A	115.6	C12—C13—H13A	119.1
C8—C7—C6	104.5 (4)	C13—C14—C1	119.7 (4)
C8—C7—H7A	110.9	C13—C14—Br2	118.1 (3)
C6—C7—H7A	110.9	C1—C14—Br2	122.1 (4)
C8—C7—H7B	110.9	C6—C15—C9	94.3 (4)
C6—C7—H7B	110.9	C6—C15—H15A	112.9
H7A—C7—H7B	108.9	C9—C15—H15A	112.9
C7—C8—C9	102.8 (4)	C6—C15—H15B	112.9
C7—C8—H8A	111.2	C9—C15—H15B	112.9
C9—C8—H8A	111.2	H15A—C15—H15B	110.3
			
C14—C1—C2—C3	0.5 (7)	C8—C9—C10—C5	−71.4 (5)
Br1—C1—C2—C3	178.0 (3)	C15—C9—C10—C5	32.6 (5)
C1—C2—C3—C12	−2.8 (6)	C5—C10—C11—C12	−0.6 (7)
C1—C2—C3—C4	176.3 (4)	C9—C10—C11—C12	−175.1 (5)
C12—C3—C4—C5	0.8 (6)	C10—C11—C12—C3	−1.5 (6)
C2—C3—C4—C5	−178.2 (4)	C10—C11—C12—C13	176.2 (4)
C3—C4—C5—C10	−2.9 (6)	C4—C3—C12—C11	1.4 (6)
C3—C4—C5—C6	173.9 (4)	C2—C3—C12—C11	−179.5 (4)
C4—C5—C6—C15	147.7 (5)	C4—C3—C12—C13	−176.3 (4)
C10—C5—C6—C15	−35.1 (5)	C2—C3—C12—C13	2.8 (6)
C4—C5—C6—C7	−107.8 (6)	C11—C12—C13—C14	−178.3 (4)
C10—C5—C6—C7	69.4 (5)	C3—C12—C13—C14	−0.6 (6)
C5—C6—C7—C8	−68.1 (5)	C12—C13—C14—C1	−1.7 (7)
C15—C6—C7—C8	36.9 (5)	C12—C13—C14—Br2	177.3 (3)
C6—C7—C8—C9	−1.0 (5)	C2—C1—C14—C13	1.8 (6)
C7—C8—C9—C10	69.5 (5)	Br1—C1—C14—C13	−175.7 (3)
C7—C8—C9—C15	−34.6 (5)	C2—C1—C14—Br2	−177.1 (3)
C4—C5—C10—C11	2.9 (7)	Br1—C1—C14—Br2	5.3 (5)
C6—C5—C10—C11	−174.7 (4)	C5—C6—C15—C9	52.5 (4)
C4—C5—C10—C9	178.7 (4)	C7—C6—C15—C9	−56.7 (4)
C6—C5—C10—C9	1.0 (5)	C10—C9—C15—C6	−51.5 (4)
C8—C9—C10—C11	103.6 (6)	C8—C9—C15—C6	56.3 (4)
C15—C9—C10—C11	−152.3 (5)		

### Hydrogen-bond geometry (Å, °)

**Table:**

*D*—H···*A*	*D*—H	H···*A*	*D*···*A*	*D*—H···*A*
C8—H8A···Br2^i^	0.97	3.00 (1)	3.843 (16)	146 (1)

Symmetry codes: (i) *x*+1/2, *y*+1/2, *z*.
